# Seroprevalence of *Toxoplasma gondii* and *Neospora caninum* in camels recently imported to Egypt from Sudan and a global systematic review

**DOI:** 10.3389/fcimb.2022.1042279

**Published:** 2022-11-14

**Authors:** Ragab M. Fereig, Hanan H. Abdelbaky, El-Sayed El-Alfy, Mohamed El-Diasty, Ahmed Elsayed, Hassan Y. A. H. Mahmoud, Alsagher O. Ali, Abdulrahman Ahmed, Ehab Mossaad, Abdullah F. Alsayeqh, Caroline F. Frey

**Affiliations:** ^1^ Division of Internal Medicine, Department of Animal Medicine, Faculty of Veterinary Medicine, South Valley University, Qena, Egypt; ^2^ Doctor of Veterinary Sciences, Veterinary Clinic, Eterinary Directorate, Qena, Egypt; ^3^ Department of Parasitology, Faculty of Veterinary Medicine, Mansoura University, Mansoura, Egypt; ^4^ Agricultural Research Center (ARC), Animal Health Research Institute-Mansoura Provincial Lab, (AHRI-Mansoura), Cairo, Egypt; ^5^ Agricultural Research Center (ARC), Animal Health Research Institute-Al Shalateen Provincial Lab (AHRI-Al Shalateen), Giza, Cairo, Egypt; ^6^ Division of Infectious Diseases, Department of Animal Medicine, Faculty of Veterinary Medicine, South Valley University, Qena, Egypt; ^7^ Department of Pathology, Parasitology and Microbiology, College of Veterinary Medicine, Sudan University of Science and Technology, Khartoum, Sudan; ^8^ Department of Veterinary Medicine, College of Agriculture and Veterinary Medicine, Qassim University, Buraidah, Qassim, Saudi Arabia; ^9^ Institute of Parasitology, Department of Infectious Diseases and Pathobiology, Vetsuisse-Faculty, University of Bern, Bern, Switzerland

**Keywords:** toxoplasmosis, neosporosis, camel, dromedary, ELISA, Egypt

## Abstract

**Introduction:**

Toxoplasma gondii and Neospora caninum are closely related intracellular protozoan parasites of medical and veterinary concern by causing abortions and systemic illness. Limited or ambiguous data on the prevalence of T. gondii and N. caninum in camels triggered us to conduct this study.

**Methods:**

Camels (n = 460) recently imported from Sudan and destined mainly for human consumption, were tested for specific antibodies against these protozoans using commercially available ELISAs. From the two only quarantine stations for camels from Sudan, 368 camels were sampled between November 2015 and March 2016 in Shalateen, Red Sea governorate, and 92 samples were collected between September 2018 and March 2021 from Abu Simbel, Aswan governorate.

**Results & Discussion:**

Overall, seropositive rates in camels were 25.7%, 3.9% and 0.8% for T. gondii, N. caninum and mixed infection, respectively. However, marked differences were found between the two study sites and/or the two sampling periods: For T. gondii, a higher rate of infection was recorded in the Red Sea samples (31.5%, 116/368; odds ratio 20.7, 5.0-85.6; P<0.0001) than in those collected in Aswan (2.2%, 2/92). The opposite was found for N. caninum with a lower rate of infection in the Red Sea samples (0.82%, 3/368; odds ratio 23.7, 6.7-83.9; P<0.0001) than in the samples from Aswan (16.3%, 15/92). Additionally, our systematic review revealed that the overall published seroprevalence of T. gondii and N. caninum was 28.6% and 14.3% in camels worldwide, respectively. To the best of our knowledge, this study provides the first record of seroprevalence of both T. gondii and N. caninum in recently imported camels kept under quarantine conditions before delivery to other Egyptian cities and regions. In addition, our review provides inclusive data on the prevalence of T. gondii and N. caninum in camel globally. This knowledge provides basic data for the implementation of strategies and control measures against neosporosis and toxoplasmosis.

## Introduction

Globally, the population size of large camelids (dromedary, *Camelus dromedarius*, and Bactrian camel, *C. bactrianus*) is estimated at over 35.5 million heads; dromedaries constitute 95% of them with the largest populations being reared in Africa and the Middle East (Zhu et al., 2019; FAOSTAT, 2020; Faye, 2020; Khalafalla and Hussein, 2021). Camels are vital to many countries’ economies, primarily those in the Arabian Peninsula, Sudan, Somalia and Ethiopia, wherein they are being used to produce milk, meat, wool, and hides, and as draught and racing animals (Zarrin et al., 2020; Khalafalla and Hussein, 2021). However, camels have been well documented to transmit a number of zoonotic diseases to humans, among others the protozoan parasite *Toxoplasma gondii* (Sazmand et al., 2019; Zhu et al., 2019; Hughes and Anderson, 2020; Mohammadpour et al., 2020). Transmission of *T. gondii* to humans may occur by eating raw or undercooked camel meat or offal, such as the liver, which is widely consumed by pastoralists (Gebremedhin et al., 2014). Another source of transmission might be unpasteurized camel milk (Boughattas, 2017; Sazmand et al., 2019), which is consumed for its higher vitamin C and iron content than cow’s milk, and for attributed important therapeutic effects for type 1 diabetes as well as allergy reduction in children.

*Toxoplasma gondii* and *Neospora caninum* are obligate intracellular protozoan parasites that infect a wide variety of domestic and wild animals as well as humans in case of *T. gondii* (Dubey, 2003; Dubey et al., 2007; Dubey, 2010). *Toxoplasma gondii* affects most warm-blooded animals and is implicated in abortion cases in women, ewes and sows. Similarly, *N. caninum* is an abortifacient agent in many mammalian species, particularly in cattle. Natural *T. gondii* and/or *N. caninum* infections of livestock are mainly acquired through the consumption of oocysts contaminating food and/or water (Elamin et al., 1992; Dubey, 2003; Dubey et al., 2007; Moore and Venturini, 2018), or through intrauterine infection.

Clinical and congenital toxoplasmosis in camels is limited to a few reports and likely underestimated in dromedaries; clinical manifestation was described as hemorrhagic enterocolitis and toxoplasmic peritonitis (Hagemoser et al., 1990; Riley et al., 2017; Sazmand et al., 2019), and more recently, abortion related to *T. gondii* was documented in a Bactrian Camel (Komnenou et al., 2022). Despite instances of anti-*N. caninum* antibodies in camels’ sera, clinical illness in large camelids has not yet been reported (Sazmand and Joachim, 2017).

In Egypt, some reports have investigated the seroprevalence of *T. gondii* and *N. caninum* in camels using different serological tests and on animals selected from different regions with special interest for those in Greater Cairo and Nile Delta regions (reviewed by Rouatbi et al., 2019; Abbas et al., 2020). Reported seroprevalence rates varied widely between 3.3% and 96.4% (Kuraa and Malek, 2016; Saad et al., 2018). Only two studies detected anti-*N. caninum* antibodies in camels in Egypt so far; Hilali et al. (1998) in Cairo using *Neospora* agglutination test found a seroprevalence of 3.7%, while Selim and Abdelhady (2020) in various Egyptian governorates using ELISA determined 11% seropositive animals, respectively. Nothing is known about the seroprevalence of these protozoans in camels imported to Egypt and destined for human consumption.

The global pooled seroprevalence of *T. gondii* infection in the Camelidae family was found to be 28.16% by a meta-analysis based on 42 studies that included large camelids (dromedary and Bactrian camels) and small camelids (guanaco, llamas, vicunas, and alpacas) (Maspi et al., 2021). As there was no particular focus on large camelids, and as some articles on *T. gondii* seroprevalence have been published in regional journals, we performed a systematic search using different databases for a comprehensive assessment of infections. Furthermore, there was no literature review of *N. caninum* prevalence in camels. Thus, we aimed to review the studies conducted on *T. gondii* and *N. caninum* infections in large camelids globally. This work had thus two aims: First, to establish the seroprevalence of *T. gondii* and *N. caninum* in recently imported camels from Sudan and kept at Shalateen quarantine, Red Sea governorate and Abu Simbel quarantine, Aswan governorate, Southern Egypt. Second, to conduct a systematic review including all published prevalence and genotyping data in large camels worldwide. The extending cross-comparisons between our results and resources from Egyptian and global studies can be used to address this serious public health issue in order to better understand the parasite epidemiology in large camelids.

## Materials and methods

### Ethical statement

This study was conducted according to instructions established by the “Research Board” of the Faculty of Veterinary Medicine, South Valley University, Qena, Egypt. The protocols were approved by Research Code of Ethics at South Valley University number 36 (RCOE-36). Blood samples were collected by a group of highly trained veterinarians and staff after consultation with the officials and animal owners.

### Animal population and geographic locations

**Figure 1:**
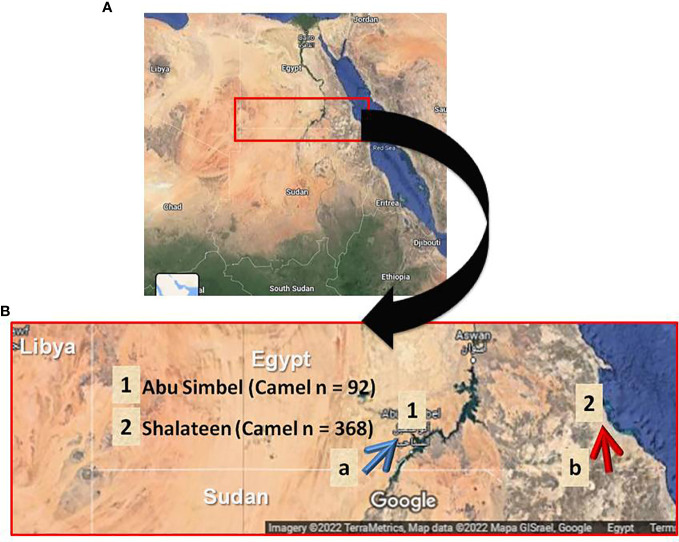
Geographical location of sample collection. **(A)** Map of Egypt illustrating the collection sites for camel samples in Southern Egypt. **(B)** Landscape enlarged area of testing samples. Blue arrow indicates the journey route of camel herds after crossing the Sudanese-Egyptian border at Argeen checking point (a) until reaching Abu Simbel quarantine (1). Red arrow shows the journey path of camel herds after crossing the Sudanese-Egyptian border at Ras Hadarba checking point (b) until reach Shalateen quarantine (2).

A total of 460 blood samples were randomly collected from recently imported camels at the only two Egyptian quarantine stations for camels imported from Sudan. Shalateen quarantine station belongs to the Red Sea governorate and is situated in southeastern Egypt, while Abu Simbel quarantine station, Aswan governorate, is situated in central south Egypt (**Figure 1**). Camels arriving at these quarantine points are usually imported in a way known as Dabuka journey, in which about 100-200 camels led by an expert man are walking for several days through the Sudanese and Egyptian desert. These camels are usually collected from different regions in Sudan, with camels of Eastern Sudan arriving at Shalateen and those of Western Sudan arriving at Abu Simbel (**Figure 1B**). At the Egyptian-Sudanese border, camels pass Argeen port before being sent to Abu Simbel, or Ras Hadarba port before arriving in Shalateen, respectively, where they are quarantined for 14 days or less (**Figure 1B**). During this period, camels are routinely checked for Rift valley fever and Corona virus infections by specific laboratory tests, and examined for apparent clinical abnormalities before permitting entrance to different Egyptian cities. Screening for *T. gondii* or *N. caninum* infection is not part of the mandated protocol. Most if not all of the imported camels are adult males, and they are primarily destined for human consumption and some for use as transport animals. In Shalateen, 368 samples were collected from November 2015 to March 2016 with two separate visits, one from November to December 2015 (n = 100 samples) and another from February to March 2016 (n = 268 samples). In Abu Simbel, 92 samples were collected from September 2018 to March 2021.

### Serum sample collection and preparation

Blood samples were collected *via* puncture of the jugular vein using glass tubes without anticoagulant. All blood samples were kept in an icebox during transportation until separation of serum at Shalateen Laboratory for those collected at Shalateen quarantine, and our laboratory at South Valley University for those collected from Abu Simbel. Serum samples from Shalateen laboratory were sent in an icebox to our laboratory and all samples were stored at −20°C at the Faculty of Veterinary Medicine, South Valley University, Qena, until use in ELISA testing.

### ELISA testing and interpretation of results

Serum samples of camels were tested for anti-*T. gondii* and anti-*N*. *caninum* antibodies, respectively, using commercial Multi-species ELISA kits (ID Screen^®^ Toxoplasmosis Indirect Multi-species and ID Screen^®^ *Neospora caninum* Competition, both ID Vet, Grables, France). Positive and negative control sera were provided in the kits and the tests were done following the manufacturer’s instructions. The optical density (OD) of ELISA results was read at 450 nm measured with an Infinite^®^ F50/Robotic ELISA reader (Tecan Group Ltd., Männedorf, Switzerland).

The Toxoplasmosis kit detects specific immunoglobulin G (IgG) antibodies against the P30 *T. gondii* protein using a peroxidase-conjugated anti-multi-species secondary antibody. The percentage sample (*S*) to positive (*P*) ratio (S/P %) for each of the test samples was calculated according to the following formula:


$$S/P\text{\% }\frac{\text{OD sample}-\text{OD negative control}}{\text{OD positive control}-\text{OD negative control }}\times 100$$

The samples with *S*/*P*% values greater than 50% were considered to be positive, those between 40 and 50% were classified as doubtful, and measurements less than or equal to 40% were considered to be negative as per the manufacturer.

The *N. caninum* kit detects specific antibodies against a purified *N. caninum* extract, using an anti-*N. caninum*- peroxidase-conjugated competing antibody. The percentage sample (*S*) to negative (*N*) ratio (S/N %) for each of the test samples was calculated according to the following formula:


$$S/N\text{\% }\frac{\text{OD sample}}{\text{OD negative control }}\times 100$$

The samples with *S*/*N*% values less than or equal to 50% were considered to be positive, those greater than 50% and less than or equal to 60% were classified as doubtful, and measurements greater than 60% were considered to be negative as per the manufacturer.

### Statistical analysis

The significance of the differences in the prevalence rates was analyzed with Chi-square (Pearson) test, 95% confidence intervals (including continuity correction) and odds ratios using an online statistical website www.vassarstats.net (accession dates; 01-02 July, 2022) as described previously (Fereig et al., 2016a; Fereig et al., 2016b). *P*-values and odds ratio were confirmed also with GraphPad Prism version 5 (GraphPad Software Inc., La Jolla, CA, USA). The results were considered significant when the *p*-value was< 0.05 (*) or highly significant when *p*-value was< 0.0001 (**).

### Data searching strategy

PubMed, Scopus, Web of Science, ScienceDirect, and Google Scholar were searched for studies on camel toxoplasmosis and neosporosis published in English up to 2022 (May, 2022). In addition, the Egyptian knowledge bank’s website (http://www.ekb.eg) was searched to collect papers from Egypt published in local journals. *Toxoplasma gondii* and *Neospora caninum* were used as search terms, along with the keyword “camel(s).” Studies were considered eligible for inclusion if they found positive samples for toxoplasmosis and neosporosis in both the one-humped dromedary camels (*Camelus dromedarius*) and the two-humped Bactrian camels (*Camelus bactrianus*).

Articles on both serodiagnosis and molecular investigations of either parasite using serum, milk and meat samples were eligible. Data from eligible studies on infections of camels was organized in a database, and the following information was extracted: sub-region/country, sample size, number of positives (%), detection methods, study year (date of samples collection), cut-off values, genetic markers and revealed genotypes (where recorded), and references with publication date. Different serological tests were included to study the prevalence of both parasites. Even in a single article, two tests may have been used, all of which were included in our literature review. Studies with more than one test were also combined with others after selection of the test with highest number of positives for estimating the pooled prevalence of both parasites either in Egypt or worldwide.

## Results

### Seroprevalence for *T. gondii* and *N. caninum* infection in camels imported to Egypt

In this study, specific antibodies against *T. gondii* were detected in 118 of the 460 surveyed animals (25.7%; 95% CI: 21.8-29.9). Consistently, 18 camels tested positive for *N. caninum* antibodies (3.9%; 95% CI: 2.4-6.2), and mixed infection was determined in 3 animals (0.65%; 95% CI: 0.17-2.1) (**Table 1**).

**Table 1:** Seroprevalence of *Toxoplasma gondii, Neospora caninum* and mixed infection in camels in Egypt.

Type of infection	No. of tested	No. of negative (%)	No. of doubtful (%)	No. of positive (%)	95% CI*
*T. gondii*	460	332 (72.2)	10 (2.2)	118 (25.7)	21.8-29.9
*N. caninum*	460	438 (95.2)	4 (0.87)	18 (3.9)	2.4-6.2
Mixed infection	460	457 (99.4)	0	3 (0.65)	0.17-2.1

* 95% CI calculated according to method described by (http://vassarstats.net/).

Based on available data, the location and period of sample collection were identified to have a significant influence on the presence of *T. gondii* and *N. caninum* antibodies in recently imported camels in Egypt. A significantly higher seroprevalence rate for *T. gondii* was recorded in animals sampled at Shalateen Quarantine, Red Sea governorate (31.5%; odds ratio = 20.7; *P* =<0.0001) compared to camel samples collected at Abu Simbel Quarantine, Aswan governorate (2.2%). Samples in Shalateen were collected between November to December 2015 and between February to March 2016, and those in Aswan between January 2018 to January 2021. Thus, the same effect was seen when univariable analysis of period of sample collection was performed. Samples collected between November to December 2015 and between February to March 2016 showed higher seropositive rates (27%; OR = 16.6; *P* =<0.0001, and 33.2%; OR = 26.3; *P* =<0.0001), respectively) than those collected between Jan 2018 to Jan 2021 (2.2%) set as a reference group (**Table 2**).

**Table 2:** Factors influencing anti-*Toxoplasma gondii* antibodies in camels in Egypt.

Analyzed factor	No. of tested	No. of negative (%)	No. of positive (%)	OR (95% CI)^#^	*P-*value^x^
**Collection region** Shalateen (Red Sea) Abu Simbel (Aswan)	368 92	252 (68.5) 90 (97.8)	116 (31.5) 2 (2.2)	20.7 (5.0-85.6) Ref	<0.0001** Ref
**Collection time** Nov 2015 – Dec 2015 Feb 2016 – Mar 2016 Sep 2018 – Mar 2021	100 268 92	73 (73) 152 (56.7) 90 (97.8)	27 (27) 89 (33.2) 2 (2.2)	16.6 (3.8-72.3) 26.3 (6.3-109.6) Ref	<0.0001** <0.0001** Ref

# Odds ratio at 95% confidence interval as calculated by http://vassarstats.net/.

**^x^** P value was evaluated by Chi square test (Pearson test) using online statistics software http://vassarstats.net/ and GraphPad Prism version 5.

** The result is significant at P< 0.0001.

Ref.; value used as a reference.

In case of *N. caninum* in camels, the seroprevalence rate recorded in animals sampled at Shalateen quarantine (0.82%; OR = 23.7; *P* =<0.0001) was significantly lower than that reported in camel samples collected at Abu Simbel Quarantine (16.3%). Again, this was also reflected when the collection periods were compared. The seropositive rates of samples collected between November to December 2015 and between February to March 2016 were lower (1%; OR = 19.3; *P* = 0.00013, and 0.7%; OR = 25.9; *P* =<0.0001), respectively) than in the samples collected between September 2018 to March 2021 (16.3%) set as a reference group (**Table 3**).

**Table 3:** Factors influencing anti-*Neospora caninum* antibodies in camels in Egypt.

Analyzed factor	No. of tested	No. of negative (%)	No. of positive (%)	OR (95% CI)^#^	*P-*value^x^
**Collection region** Shalateen (Red Sea) Abu Simbel (Aswan)	368 92	365 (68.5) 77 (97.8)	3 (0.82) 15 (16.3)	23.7 (6.7-83.9) Ref	<0.0001** Ref
**Collection time** Nov 2015 – Dec 2015 Feb 2016 – Mar 2016 Sep 2018 - Mar 2021	100 268 92	99 (99) 266 (99.3) 77 (83.7)	1 (1) 2 (0.7) 15 (16.3)	19.3 (2.5-149.2) 25.9 (5.8-115.8) Ref	0.00013* <0.0001** Ref

# Odds ratio at 95% confidence interval as calculated using http://vassarstats.net/.

**^x^** P value was evaluated by Chi square test (Pearson test) using online statistics software http://vassarstats.net/ and GraphPad Prism version 5.

* The result is significant at P < 0.05.

**The result is significant at P < 0.0001.Ref.; value used as a reference.

### Global systematic review data

A total of 79 studies were included and reviewed in this article comprising 74 articles on large camels’ toxoplasmosis and 14 articles conducted on neosporosis, respectively, of which 9 articles investigated both parasites. For Egypt, a pooled prevalence rate of 38.5% for antibodies against *T. gondii* was found in 1,444 serum samples of dromedaries collected from various governorates and tested with various assays (**Table 4**). Furthermore, 71 milk samples from camels tested for *T. gondii* antibodies revealed a pooled prevalence of 18.3% in this matrix (**Table 4**). For antibodies against *N. caninum*, a pooled prevalence of 8.4% was found in 443 serum samples (**Table 5**). Globally, 12,092 serum samples collected from large camels were investigated for *T. gondii* antibodies, of which 3,457 were found to be positive giving an estimated overall prevalence of 28.6% (**Table 4**). Meanwhile, 2,654 serum samples were investigated for *N. caninum* antibodies, of which 380 samples were positive, resulting in an estimated pooled prevalence of 14.3% (**Table 5**). *Toxoplasma gondii* type I, II, and III were identified in meat, blood and milk samples from camels using different molecular markers (**Table 6**).

**Table 4:** Seroprevalence of anti-*Toxoplasma gondii* antibodies in camels (*Camelus dromedarius* and *Camelus bactrianus*) worldwide.

Country	Region	Study Year	No. tested	No. positive (%)	Diagnostic methods	Cut-off	Reference
Afghanistan*	Kabul	1974	19	14 (73.7)	IHA	1:64	Kozojed et al. (1976)
Algeria	Biskra, El- Oued, Ouargla, and Ghardaia	2018	320	48 (15)	ELISA	MI	Abdallah et al. (2020)
China^©^	Qinghai	2010–2011	234	7 (2.99)	IHA	1:64	Wang et al. (2013)
Czech Republic^©^	–	2001–2011	36	22 (61) 25 (69)	IFAT ELISA	1:50 MI	Bártová et al. (2017)
Egypt	Different	–	49	3 (6.1)	IFA		Maronpot and Botros (1972) ^a^
	Ismailia	–	43	29 (67.4)	DT	1:8	Rifaat et al. (1977) ^a^
	Assiut	–	80	12 (15.0)	DT	1:16	Michael et al. (1977) ^a^
	Menoufiya	–	80	15 (18.7)			
	Matrouh	–	80	40 (50.0)			
	Menoufiya	–	30	17 (56.7)	DT	1:8	Rifaat et al. (1978) ^a^
	Assiut	–	119	30 (24.4)	DT	1:4	Fahmy et al. (1979) ^a^
	Sharkia	–	19	5 (26.3)	IHA		El-Ridi et al. (1990) ^a^
	Gharbia	–	36	6 (16.7)	IHA	1:64	Ibrahim et al. (1997) ^a^
	Cairo	NS	166	29 (17.4)	MAT	1:25	Hilali et al. (1998)
	Cairo	NS	150	^1^ 27 (18.0), ^2^ 30 (20.0), ^3^ 46 (30.7), ^4^ 41 (27.3)	MAT#	1:25	Shaapan and Khalil (2008)
	Cairo	NS	60	40 (66.7)	ELISA^¥^	NS	Toaleb et al. (2013)
	Assiut	2014-2016	56	20 (35.7) 54 (96.4)	LAT ELISA	1:2 MI	Kuraa and Malek (2016)
	Cairo, Giza	NS	34	9 (26.5)	ELISA	NS	Elfadaly et al. (2017)
	Mersa Matrooh	2014	53	32 (60.37)	LAT	MI	Osman et al. (2016)
	Qalyubia	2014-2015	120	6 (5) 63 (52.5)	IHA iELISA	MI	Ahmed et al. (2017)
	Upper Egypt	NS	30-Milk	1 (3.33)	ELISA	MI	Saad et al. (2018)
	Aswan	2017	37	12 (32.4)	LAT	MI	Sameeh et al. (2021)
	Matrouh	2016-2017	124	80 (64.51)	ELISA	MI	Khattab et al. (2022)
	Beni Suef, Giza, Monufa, Alexandria, Sharqia, Matruh, and Faiyum	2019-2021	108 41-Milk	34 (31.48) 12 (29.26)	ELISA	MI	Zeedan et al. (2022)
Ethiopia	Fentale	2012-2013	455^b^ 451^b^	220 (49.62) 179 (40.49)	DAT iELISA	1:40 MI	Gebremedhin et al. (2014)
	Afar	NS	384	262 (72.9)	MAT	1:40	Hadush et al. (2015)
	Borana	2013-2014	396	33 (8.33)	DAT	1:40	Gebremedhin et al. (2016)
	Oromia	2011-2013	292	42 (14.38)	iELISA	MI	Tilahun et al. (2018)
India	Rajasthan	NS	108 231	12 (11.1) 25 (10.8)	SFDT IHA	1:4 1:16	Gill and Prakash (1969)
Iraq	Al-Najaf	2011-2012	360 91	91 (25.2) 15 (16.4)	LAT ELISA	MI	Mahmoud et al. (2014)
	Wasit	NS	92	19 (20.6)	ELISA	NS	Asal and Al Zubaidy (2016)
	Al-Najaf	2014-2015	227 70	70 (30.8) 16 (22.8)	LAT iELISA	MI	Al-Dhalimy and Mahmoud (2019)
	Kirkuk	2018	76	20(26.3)	SFDT	1:16	Yawoz et al. (2021)
Iran	Mashhad	2004-2005	120	5 (4.16)	IFAT	1:20	Sadrebazzaz et al. (2006)
	Isfahan	–	310	87 (28.06)	IFAT	1:16	Hosseininejad et al. (2010)
	Tehran, Isfahan, and Fars	2011-2012	160-Milk	3 (1.87)	cELISA	–	Dehkordi et al. (2013)
	Yazd	2008-2009	254	37 (14.56)	MAT	1:20	Hamidinejat et al. (2013)
	southern provinces	2013-2014	493	49 (9.93)	ELISA	MI	Azma et al. (2015)
	Kerman, Razavi Khorasan, and south Khorasan	2015	50	13 (26)	MAT	1:20	Tavakoli Kareshk et al. (2018)
Italy	Southern	2014	9**^©^** 5	2 (22) 2 (40)	IFAT	1:50	Marková et al. (2019)
Nigeria	Kano	NS	159	0 (0)	IHA	1:64	Okoh et al. (1981)
Pakistan	Bahawalpur	NS	100	10 (10)	LAT	1:16	Chaudhry et al. (2014)
	Punjab	2015	201	36 (17.91)	LAT	MI	Lashari et al. (2018)
	Punjab	2016	897	360 (40.1)	iELISA	1:100	Fatima et al. (2019)
	Mianwali	2017-2018	350	133 (38.0)	iELISA	NS	Shehzad et al. (2022)
Saudi Arabia	–	NS	46	0	IHA	1:64	Hossain et al. (1987)
	–	NS	227	36 (16)	IHA	1:64	Hussein et al. (1988)
	Riyadh	2010	412	27 (6.5%)	IFAT	1:20	Al-Anazi (2011)
	Al-Riyadh, Alharig, Al- Solyl, Dar- maa and Wady Al- Dawaser	2009-2010	482	219 (45.44)	iELISA	MI	Al-Khatib (2011)
	Al-Ahsa	2010	210	17 (8)	ELISA	MI	Al-Mohammed (2011)
	Ad-Dawadimi, Shaqra, Afif, Al-Quwayiyah	NS	713	94 (13.1)	LAT	1:8	Al-Anazi (2012)
	Riyadh	NS	182	43 (23.6)	IFAT	1:32	Alanazi (2013)
	Najran	2014	90	22 (24.4) 19 (21.1)	IHA ELISA	1:80 1:80	Mosa et al. (2015)
	Qassim	NS	141	0 (0)	ELISA	MI	Derar et al. (2017)
	Hofuf, Riyadh, Tabuk, Jizan, Taif	NS	199	68 (34.2)	ELISA		Mohammed et al. (2020)
Somalia	Benadir	NS	64	4 (6.3)	LAT	1:2	Kadle (2014)
Spain	Canary Islands	2012	96	35 (36.5)	MAT	1:25	Mentaberre et al. (2013)
Sudan	Kordofan and central regions	1982-1983	204	111 (54.4)	IHA	1:40	Zain Eldin et al. (1985)
	Tamboul and Butana plains	NS	95	11 (11.57)	IHA	1:64	Abbas et al. (1987)
	Tampoul	NS	102	23 (22.5)	MSF	1:5	Bornstein and Musa (1987)
	Butana plains	NS	482	323 (67)	LAT	1:8	Elamin et al. (1992)
	Butana area, north and south Kordofan	NS	153	34 (22.2)	LAT	1:4	Khalil et al. (2007)
	Khartoum	NS	70	14 (20)	LAT	1:8	Khalil and Elrayah (2011)
	Tumbool	2009	100	44 (44)	LAT	NS	Basheir et al. (2012)
	Khartoum	2012-2014	61	33 (54.1)	LAT	1:2	Ibrahim et al. (2014); Ibrahim et al. (2015)
	Tamboul	NS	150	47 (31.3)	LAT	NS	Elias et al. (2017)
	West Kordofan, and Blue Nile states	NS	45	6 (13.3)	LAT	1:32	Abdelbaset et al. (2020)
Turkey	Nevsehir	2010	11	10 (90.9)	SFDT	1:16	Utuk et al. (2012)
UAE	Abu Dhabi	NS	97 143	30 (30.9) 52 (36.4)	DAT IHA	1:24 1:220	Afzal and Sakkir (1994)
	NS	NS	100	18 (18)	LAT	1:64	Chaudhary et al. (1996)

ELISA, enzyme-linked immunosorbent assay; DAT, direct agglutination test; IHA, indirect hemagglutination; LAT, latex agglutination test; MAT, modified agglutination test; MSF, modified Sabin-Feldman dye test; SFDT, Sabin-Feldman Dye Test; cELISA, Capture Enzyme-Linked Immunosorbent Assay; MI; Data results interpretations were done according to Manufacturer’s Instructions; NS, not stated.

a Studies were reviewed by Abbas et al., 2020.

b Samples were from the same animals but different sample size for the two used tests.

* The species of camels were not indicated.

# MAT was conducted using formalin-treated whole tachyzoites from different antigen; ^1^ RH strain, ^2^ local equine strain, ^3^ local camel strain and ^4^ local sheep strain.

^¥^ ELISA using T. gondii camel strain isolated fraction.

^©^ Bactrian camels.

**Table 5:** Seroprevalence of anti-*Neospora caninum* antibodies in camels (*Camelus dromedarius* and *Camelus bactrianus*) worldwide.

Country	Region	Study Year	No. tested	No. positive (%)	Diagnostic methods	Cut-off	Reference
Czech Republic	–	2001–2011	36	17 (47) 11 (31)	IFAT cELISA	1:50 MI	Bártová et al. (2017)
Egypt	Cairo	NS	161	6 (3.7)	NAT	1:40	Hilali et al. (1998)
	Red Sea, Qalyubia, Kafr ElSheikh	2018–2019	282	31 (11)	ELISA	MI	Selim and Abdelhady (2020)
Iran	Mashhad	2004-2005	120	7 (5.83)	IFAT	1:20	Sadrebazzaz et al. (2006)
	Isfahan	2008	310	10 (3.22)	IFAT	1:50	Hosseininejad et al. (2009)
	Yazd	2008-2009	254	10 (3.94)	NAT	1:20	Hamidinejat et al. (2013)
	Bushehr	NS	92	25 (27)	NAT	1:20	Namavari et al. (2017)
Italy	Southern	2014	9**^©^** 5	0 (0) 0 (0)	IFAT	1:50	Marková et al. (2019)
Pakistan	Punjab	2014-2015	81	9 (11.1)	cELISA	MI	Nazir et al. (2017)
Spain	Canary Islands	2012	100	86 (86)	cELISA	MI	Mentaberre et al. (2013)
Saudi Arabia	Riyadh	2010	412	23 (5.6)	IFAT	1:20	Al-Anazi (2011)
	variable	2013	532	117 (21.99)	ELISA	MI	Aljumaah et al. (2018)
	Hofuf, Riyadh, Tabuk, Jizan, Taif	NS	199	33 (16.6)	ELISA	MI	Mohammed et al. (2020)
Sudan	Khartoum	2013-2014	61	6 (9.8)	cELISA	NS	Ibrahim et al. (2015)

ELISA, enzyme-linked immunosorbent assay; cELISA, competitive ELISA; IFAT, indirect fluorescent antibody test; NAT, Neospora agglutination test; MI; Data results interpretations were done according to Manufacturer’s Instructions; NS, not stated.

**Table 6:** Summary of molecular detection and genotyping reports for *T. gondii* and *N. caninum* infecting camels worldwide.

Country	Region	Type of samples	Study Year	Marker#	No. tested	No. positive (%)	Isolates (no.)	Genotyping method	Protozoan species/Remarks	Reference
Egypt	Qalyubia	Blood	2014-2015	NC-5	50	12 (24)	4	–	–	Ahmed et al. (2017)
	Cairo, Giza	Diaphragm and thigh muscles	NS	B1	9	5 (55.5)	2	PCR-RFLP (5′-SAG2, 3′-SAG2)	*T. gondii* (type II, III)	Elfadaly et al. (2017)
	Cairo	Cardiac muscles	2017-2018	B1	90	1 (1.1)	1	PCR-RFLP (5′-SAG2, 3′-SAG2, alt. SAG2)	*T. gondii* (type II)	El-Alfy et al. (2019)
	Aswan	Meat samples	2017	B1	12	6 (50)	–	–	–	Sameeh et al. (2021)
	Matrouh	Buffy coat	2016-2017	B1 & P30	80	–	1	–	–	Khattab et al. (2022)
	7 provinces	Blood Milk	2019-2021	B1	108 41	18(16.6) 2 (4.8)	–	–	–	Zeedan et al. (2022)
Iran	Tehran, Isfahan, and Fars	Milk	2011-2012	B1	160	4 (2.5)	–	–	–	Dehkordi et al. (2013)
	Isfahan	Blood	2013	18srRNA	122	8 (6.6)	–	–	–	Khamesipour et al. (2014)
	Sabzevar	Diaphragm and heart muscles	2014	B1	40	26 (65)	9	PCR-RFLP B1	*T. gondii* (type II, III)	Aliabadi and Ziaali (2016)*
	Kerman, Razavi Khorasan, and south Khorasan	Diaphragm heart muscles	2015	B1	50 50	7 (14) 6 (12)	3	PCR-RFLP (GRA6)	*T. gondii* (type I, II, III)	Tavakoli Kareshk et al. (2018)
Mongolia^©^	Tuv and Omnigovi	Milk samples	NS	ITS-1 and B1	9	5 (55.5)	4	–	–	Iacobucci et al. (2019)
UAE*	Abu Dhabi	Blood and milk	–	–	53	13	13	PCR-RFLP (SAG2)	*T. gondii* (type I, II)	Sharif et al. (2017)

# Target gene used for the pathogen detection using PCR.

* This study did not mention the camel genus (dromedary or Bactrian).

^©^Bactrian camels.

NS, not stated.

## Discussion

In the present study, we investigated the seroprevalence of *T. gondii* and *N. caninum* in camels recently imported to Egypt from Sudan. The quarantine stations of Shalateen, Red Sea governorate, and Abu Simbel, Aswan governorate, are the only gates for importing camels to Egypt coming from Sudan. These animals are quarantined and subjected to numerous veterinary examinations including clinical and laboratory procedures, but not including *T. gondii* or *N. caninum* screening. Data on the seroprevalence of these important parasites is therefore missing, which is particularly concerning as these animals from Sudan are mainly destined for human consumption. We now determined an overall seroprevalence of *T. gondii* of 25.7%, which could represent a considerable infection risk for consumers. The seroprevalence for *N. caninum* and mixed infections was much lower with 3.9% and 0.65%, respectively. These results fall within the ranges of seroprevalence established in previous serological studies in large camels for *T. gondii* and/or *N. caninum* (**Tables 4** and **5**).

Our tested camels are short-lived in Egypt, and most if not all of them are adult males as the Sudanese government restricts the export of female camels for human consumption. The dromedaries are usually transported in a way known as “Dabuka journey” which means travelling from Sudan to Egyptian border ports by a long walk. This journey usually takes several days to weeks before arriving to Egypt, and thus Sudan might be suspicious as the original country of infection. Indeed, numerous reports revealed the high prevalence and endemicity of *T. gondii* and *N. caninum* among camels in various Sudanese regions (Zain Eldin et al., 1985; Abbas et al., 1987; Bornstein and Musa, 1987; Elamin et al., 1992; Khalil et al., 2007; Ibrahim et al., 2015; Elias et al., 2017; see **Tables 4** and **5**). However, we found marked differences in the seroprevalence of *T. gondii* and *N. caninum* between the two quarantine stations. While we cannot rule out that these differences were caused by the difference in sampling years, we would argue that the different origin in Sudan and differences in travel routes of the camels in the two quarantine stations played a crucial role. Camels arriving in Shalateen made a long journey from Eastern Sudan to Southeastern Egypt where wild cats such as leopard *(Panthera pardus)* were already reported (Soultan et al., 2017). Moreover, camels from Eastern Sudan, before being exported to Egypt, are quarantined in the Sudanese government quarantine near Kassala city, around which many stray domesticated cats are usually seen. In addition, in Eastern Sudan, wild cats (Civet cat; *Civetticitis civetta* and Serval cats; *Leptailurus serval*) are abundant (Khalid, 2016). As camels are probably mainly infected by ingestion of *T. gondii* oocysts, the presence of wild and stray cats could explain the higher seroprevalence for *T. gondii* in camels from Eastern Sudan. However, more studies are needed for the detection of *Toxoplasma* oocysts in the feces of the wild and stray cats in the area. On the other hand, the higher seroprevalence for *N. caninum* recorded in the camels that have entered Egypt through Abu Simbel region, could be explained by the fact that these animals are originally from Western Sudan and owned by nomad tribes. Common animal husbandry practice in that area is the use of guard dogs (at least 10 dogs per camel herd of 100 animals). This co-herding of camels and dogs can be considered as a major risk factor for many diseases including trypanosomosis (Mossaad et al., 2017), hydatidiosis (Ibrahim et al., 2011) and also *N. caninum* infection, as seen in the current study. It remains to be investigated whether *N. caninum* may also cause abortions in dromedaries, and the prevalence of *N. caninum* in dogs in the area should also be studied. In addition, camels with free access to pasture might have a greater opportunity of ingesting *T. gondii* or *N. caninum* oocysts compared to those raised in intensive and semi-intensive breeding systems (Venturoso et al., 2021).

As we explained in our systematic review, data on seroprevalence of *T. gondii* and *N. caninum* in camels is scarce and further studies are needed whether in Egypt or other countries. Our review also revealed a significant limitation regarding the overall number of camels (n = 443) that had been tested in all previous studies for *N. caninum* which is lower than the camels tested in this study (n = 460). Our seroprevalences for *T. gondii* and *N. caninum* in camels were lower than in the calculated pooled prevalence from previous studies Egypt for *T. gondii* (556/1144, 38.5%), and *N. caninum* (37/443, 8.4%), respectively. However, our positive rate for *T. gondii* in camel was similar to that reported globally from a pooled prevalence rate (3451/12047, 28.6%) but our positive rate for *N. caninum* was lower than that estimated worldwide (380/2654, 14.3%). These variable observations can be explained by the vast differences depending on the country, region, age, sex, season, breed of the animals, and type of serological test used (Dubey and Lindsay, 2006).

In conclusion, we provide novel data on the seroprevalence of *T. gondii* and *N. caninum* in recently imported camels from Sudan, quarantined in Shalateen and Abu Simbel, Southern Egypt. Our results demonstrated a high exposure of camels to *T. gondii* and *N. caninum* infection either in Egypt or in Sudan. Also, our study revealed the substantial lack of data on camel toxoplasmosis and neosporosis in Egypt and worldwide, demonstrating the need for further studies.

## Data availability statement

The raw data supporting the conclusions of this article will be made available by the authors, without undue reservation.

## Ethics statement

The animal study was reviewed and approved by Research Code of Ethics at South Valley University number 36 (RCOE-36).

## Author contributions

Conceptualization and design: RF, CF. Experiments, formal analysis, investigation: RF, HA, E-SE-A, ME-D. Resources and shared materials: RF, HA, E-SE-A, ME-D, AE, HM, AOA, AA, EM, AA, CF. Writing—original draft, RF, HA, E-SE-A, CF. Writing—review and editing: RF, HM, AOA, EM, AA, CF. Project administration: RF, CF. All authors contributed to the article and approved the submitted version.

## Acknowledgments

We thank all veterinarians and officials who helped in collection of samples of recently imported quarantined camels and the animal owners for their cooperation in providing animals and required data and information. We appreciate the great help of our colleagues at Department of Animal Medicine, Faculty of Veterinary Medicine, South Valley University, Qena, Egypt, for their cooperation and technical assistance.

## Conflict of interest

The authors declare that the research was conducted in the absence of any commercial or financial relationships that could be construed as a potential conflict of interest.

## Publisher's note

All claims expressed in this article are solely those of the authors and do not necessarily represent those of their affiliated organizations, or those of the publisher, the editors and the reviewers. Any product that may be evaluated in this article, or claim that may be made by its manufacturer, is not guaranteed or endorsed by the publisher.
